# Cost Analysis of Screening for, Diagnosing, and Staging Prostate Cancer Based on a Systematic Review of Published Studies

**Published:** 2007-09-15

**Authors:** Donatus U Ekwueme, Leonardo A Stroud, Yanjing Chen

**Affiliations:** Division of Cancer Prevention and Control, National Center for Chronic Disease Prevention and Health Promotion, Centers for Disease Control and Prevention; Division of Cancer Prevention and Control, National Center for Chronic Disease Prevention and Health Promotion, Centers for Disease Control and Prevention, Atlanta, Georgia; Division of Cancer Prevention and Control, National Center for Chronic Disease Prevention and Health Promotion, Centers for Disease Control and Prevention, Atlanta, Georgia

## Abstract

**Introduction:**

The reported estimates of the economic costs associated with prostate cancer screening, diagnostic testing, and clinical staging are substantial. However, the resource costs (i.e., factors such as physician's time, laboratory tests, patient's time away from work) included in these estimates are unknown. We examined the resource costs for prostate cancer screening, diagnostic tests, and staging; examined how these costs differ in the United States from costs in other industrialized countries; and estimated the cost per man screened for prostate cancer, per man given a diagnostic test, and per man given a clinically staged diagnosis of this disease.

**Methods:**

We searched the electronic databases MEDLINE, EMBASE, and CINAHL for articles and reports on prostate cancer published from January 1980 through December 2003. Studies were selected according to the following criteria: the article was published in English; the full text was available for review; the study reported the resource or input cost data used to estimate the cost of prostate cancer testing, diagnosing, or clinical staging; and the study was conducted in an established market economy. We used descriptive statistics, weighted mean, and Monte Carlo simulation methods to pool and analyze the abstracted data.

**Results:**

Of 262 studies examined, 28 met our selection criteria (15 from the United States and 13 from other industrialized countries). For studies conducted in the United States, the pooled baseline resource cost was $37.23 for screening with prostate-specific antigen (PSA) and $31.77 for screening with digital rectal examination (DRE). For studies conducted in other industrialized countries, the pooled baseline resource cost was $30.92 for screening with PSA and $33.54 for DRE. For diagnostic and staging methods, the variation in the resource costs between the United States and other industrialized countries was mixed.

**Conclusion:**

Because national health resources are limited, a decision about whether to invest in early detection of prostate cancer requires an understanding of the factors included in estimates of the economic cost of this disease. This study may benefit health policy makers charged with allocating resources for prostate cancer.

## Introduction

Prostate cancer is the second most common cancer among men worldwide and the fifth most common cancer in the world (1,2). Among developed countries, the age-adjusted death rate for prostate cancer is highest in Sweden, with an estimated rate of 27.7 per 100,000 men, and lowest in Japan, with an estimated rate of 5.7 per 100,000 men (3). The United States falls between these two extremes, with an estimated rate of 15.8 per 100,000 men.

The U.S. Preventive Services Task Force (USPSTF) (www.ahrq.gov/clinic/uspstf.htm) recommends screening tests for early detection of breast, colorectal, and cervical cancers, but screening for prostate cancer remains controversial (4-6). Factors contributing to the controversy include the lack of conclusive scientific evidence demonstrating the effectiveness of screening in reducing mortality associated with prostate cancer (6) and the absence of an international consensus about routine screening (7,8). Nevertheless, screening for this disease is widespread (6). For example, in 2000, between 34% and 61% of U.S. men aged 50 years or older reported having a prostate-specific antigen (PSA) test within the previous year (9,10,11).

The reported economic costs associated with screening for prostate cancer are substantial and vary widely. For example, in 1995 Barry and colleagues estimated the cost to Medicare of first-year PSA testing for men aged 65 to 79 years as $2.1 billion (12). In 1994 Lubke and colleagues estimated the costs of a first-year national testing program using PSA and digital rectal examination (DRE) for men aged 50 to 69 years to range from $17.6 to $25.7 billion (13). In 1990 Optenberg and Thompson estimated the costs of a first-year mass screening program for men aged 50 through 74 years to range from $11 to $28 billion (14). Often, researchers do not provide the components of the resource costs (i.e., factors such as physician's time, laboratory tests, patient's time away from work) used to estimate the economic cost of prostate cancer. When resource costs are provided, they are often presented without an explanation as to the types of resources used in calculations or how these resources were measured or valued. It is not possible to determine whether the reported cost of screening includes the costs associated with patients' travel time, time off from work, loss of leisure time, transportation, physicians' consultation time, other medical staff time, medical supplies, office or room space, equipment, and patient recruitment. As a result, the costs reported from economic studies of prostate cancer vary widely.

We reviewed the published articles from 1980 to 2003 in order to summarize publicly available data on the resource costs used in estimating the economic effects of prostate cancer. These resource costs are needed to estimate the economic cost of the disease accurately. We examined the resource costs of prostate cancer screening, diagnosing, and staging; examined how resource costs differ in the United States from the costs in other countries; and estimated a cost per man screened for prostate cancer, per man given a diagnostic test, and per man given a clinically staged diagnosis of the disease.

## Methods

### Search and selection processes

We searched the following computerized electronic databases for articles published from January 1980 through December 2003: MEDLINE (1980–2003), EMBASE (1980–2003), and CINAHL (1983–2003). Our search terms included *prostatic neoplasms,* *prostate cancer, prostate-specific antigen, digital rectal examination, transrectal ultrasound, biopsy, cost, cost analysis, cost-benefit analysis, *and* economic cost*. We manually searched the bibliographies of retrieved articles and reports to find additional articles.

The following were the preset inclusion criteria for the studies selected: the article was published in English; the full text was available for review; the resource or input cost data used to estimate the cost of screening, diagnosing, or clinically staging prostate cancer were reported in the article; and the study was conducted in countries designated as established market economies as defined by the World Bank (15).

### Data extraction

From eligible articles and reports, we extracted the following data using a modified extraction method developed by the U.S. *Guide to Community Preventive Services *for reviews of economic evaluations (16): study characteristics (e.g., researcher[s], year results were published, country of study, study setting), participants' characteristics (e.g., age, population screened, number of biopsies performed, whether prostate cancer was detected), and resource cost characteristics (e.g., year of cost data used in the study, currency denomination, cost components). We extracted the resource costs provided in the articles and attempted to contact the researchers for clarification when necessary. Any uncertainty about including data from any article was resolved by consensus of all coauthors.

### Data analysis

Resource costs were separated into two categories: those found through studies done in the United States and those found through studies done in other industrialized countries. For each category, we analyzed the resource costs for three evaluation methods: screening, diagnosing, and staging.

Because our interest was only in ascertaining the resource (i.e., input) costs used in calculating the cost of each screening procedure, we identified and pooled separately the resource costs of serum PSA, free/total PSA (F/tPSA), complex PSA (cPSA), and DRE.

For diagnostic procedures, we identified and pooled separately the resource costs of transrectal ultrasound (TRUS) and biopsy. We defined TRUS as a component of diagnostic methods. Because TRUS is used when results of PSA, DRE, or both are abnormal, we assumed that TRUS serves as a diagnostic procedure to confirm the presence or absence of a prostate cancer tumor before further investigation. Biopsy procedures included core-needle, TRUS-guided, fine-needle aspiration, needle, and transrectal needle. The resource costs for performing a biopsy represented the combined resource costs of these biopsy methods. We also included the resource costs for a urology consultation, defined as any consultation or referral to a urologist, clinical oncologist, or any other specialist after abnormal test results. We reported the resource costs for each diagnostic method separately and noted that a urology consultation is a process measure, not a diagnostic method per se.

For staging methods, we identified the resource costs of clinical staging procedures and pathologic or histologic analysis of specimens. For purposes of our analysis, we included in clinical staging procedures computerized tomography (CT), magnetic resonance imaging (MRI), radionuclide bone scan, pelvic lymph node excision and analysis, and pelvic echography. We also included the resource cost of pathologic or histologic analysis of a specimen as a part of the staging method. We acknowledge that this is not a staging method but a process measure; however, these data were reported separately.

### Currency conversion methods

To allow for greater comparability among studies and countries and to standardize the resource costs to 2003 U.S. dollars, we used three conversion methods: the cost-to-charge ratio, the Consumer Price Index (CPI) for all commodities, and purchasing power parity (PPP). For the studies conducted in the United States, we used the cost-to-charge ratio to convert resource costs reported as charges (i.e., prices) into actual costs of providing health services (17,18).

We adjusted all resource costs to 2003 U.S. dollars using the CPI (i.e., measure of changes in the average price of consumer goods and services) (19). For studies conducted in other countries, we used the country-specific CPI to adjust costs to 2003 country-specific currency. For example, we used the Australian CPI to update cost to 2003 Australian dollars. The CPIs for other countries were obtained from the Organisation for Economic Co-operation and Development (20).

PPP converts currency units from other countries to U.S. dollars to eliminate differences in price levels among countries (21). Using this method, we converted currencies of other countries to U.S. dollars by multiplying the adjusted country-specific currency by the PPP rate for each country (22).

### Measurement of resource costs used

Resource categories included direct and indirect costs. Direct costs included the resources used in the early detection of prostate cancer, such as the physician's consultation time, other medical staff's time, medical supplies, office or room space, equipment, and patient recruitment. Indirect costs included the patient's loss of income from time off from work, loss of leisure time, transportation cost, and travel time. We wrote this article from the societal perspective (17); that is, all identified direct and indirect resource costs for prostate cancer prevention are taken into account, regardless of who might pay for them.

### Statistical analysis

We obtained pooled standardized resource costs by using standard descriptive statistics. We estimated the cost per man screened and given a clinically staged diagnosis of prostate cancer by using the weighted mean method (23). For screening methods, the weighted mean cost per man screened was computed by multiplying the standardized resource cost from each study by the corresponding number of men screened, summing this product, and dividing by the total number screened. This is expressed as

Cost per man screenedj=∑i=1k[(Number screened)i*(Resource cost associated with screening)]i∑i=1k(Number screened)i

where *j* = screening methods (i.e., PSA, F/tPSA, cPSA, and DRE); Σ = summation; *i* = each study; *k* = number of studies, and *number tested *= the number of men screened for prostate cancer. We used the same approach for diagnostic testing and staging methods. We computed 95% confidence intervals (CIs) for the weighted mean cost estimates.

### Sensitivity analysis

We conducted multivariate sensitivity analyses using the Monte Carlo simulation method to appraise uncertainty in the pooled resource costs (24,25). We fitted probability distributions to the resource cost data from the studies included in each evaluation method. Using the fitted distributions, we performed simulations using @Risk software (Palisade Corporation, Newfield, New York), which uses Monte Carlo sampling methods. We performed 1000 independent simulation trials. On each simulation trial, a value for each parameter was drawn from its associated distribution and stored for subsequent analysis. The results from the simulations are presented as means with 95% CIs and medians with 25th and 75th percentiles. The 95% CIs from the simulation were calculated as

Mean±Zα*SD/Number of iterations,where SD = standard deviation

## Results

### Descriptive results

**Figure 1:**
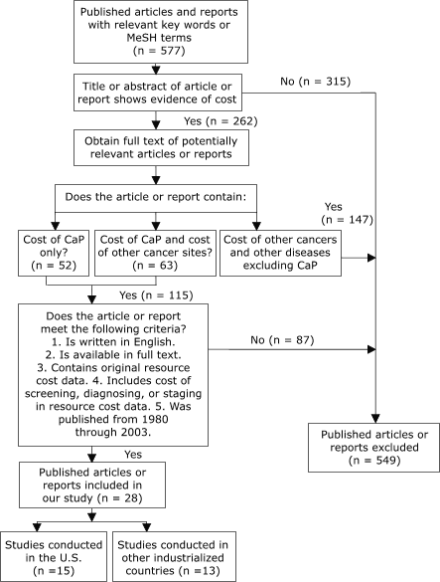
Study selection process, Cost Analysis of Screening for, Diagnosing, and Staging Prostate Cancer, 1980–2003. CaP indicates prostate cancer; MeSH, medical subject headings.

We identified 262 studies, of which 28 met all inclusion criteria (Figure 1). Among these studies, 15 (53.6%) were from the United States (14,26-39), 4 (14.3%) from Canada (40-43), 4 (14.3%) from Sweden (44-47), 3 (10.7%) from the United Kingdom (48-50), 1 (3.6%) from Australia (51), and 1 (3.6%) from Japan (52). For studies conducted in the United States, the number of men screened ranged from 564 to 19.1 million; the number of biopsies performed ranged from 23 to 3.4 million; and the participants' ages ranged from 40 to 75 years (Table 1). For studies conducted in other industrialized countries, the number of men screened ranged from 472 to 533,402; the number of biopsies performed ranged from 29 to 45,873; and the participants' ages ranged from 40 to 93 years. A summary of the standardized resource costs for each screening method is in Table 2. Among these studies, only one conducted in the United States reported the resource cost of screening with F/t PSA and cPSA (39).

**Table:** 

Figure 1 is a flow chart consisting of boxes that describe the steps in the study selection process. Selection began with a search for all published articles and reports with relevant key words or Medical Subject Headings (MeSH) terms (n=577). Of these, 262 were selected because the title or abstract of the article or report showed evidence of cost. A total of 315 were rejected as they did not include such evidence. The authors obtained full text of the 262 that contained evidence of cost. These were evaluated to determine if the article or report contained the following:
Cost of prostate cancer only (n = 52). Cost of prostate cancer and cost of other cancer sites (n = 63). Cost of other cancers and other diseases excluding prostate cancer (n = 147).
If the article contained cost of prostate cancer only or cost of prostate cancer and of other cancer sites, it was evaluated to determine if the article or report met the following criteria:
Was written in the English language. Was available in full text. Contained original resource cost data. Includes cost of screening for, diagnosing, or staging prostate cancer in resource cost data. Was published from 1980 through 2003.
Articles that met these criteria totaled 28. Of these, 15 were conducted in the United States, and 13 were conducted in other industrialized countries.
A total of 87 articles or reports were rejected for not meeting these criteria.
The total number of articles or reports that contained relevant key words or MeSH terms that were subsequently rejected totaled 549. The total number that met all criteria and were included totaled 28.

### Baseline results

For studies conducted in the United States, the pooled baseline resource cost for screening with PSA obtained from 13 studies was $37.23, and the pooled baseline resource cost for screening with DRE obtained from eight studies was $31.77 (Table 3). For studies conducted in other countries, the pooled baseline resource cost for screening with PSA from 10 studies was $30.92, and the pooled baseline resource cost for screening with DRE obtained from eight studies was $33.54.

For diagnostic tests and staging methods, the variations in the resource costs between the United States and other countries were mixed. The pooled baseline resource costs were 2.3 times higher for TRUS and 2.4 times higher for biopsy in the United States than in other countries. The pooled baseline resource cost of a urology consultation was 1.3 times higher in other countries than in the United States. For clinical staging, the pooled baseline resource cost was 2.4 times higher in the United States than in other countries, but the pooled baseline costs for pathologic or histologic specimen analysis was 1.4 times higher in other countries.

**Figure 2:**
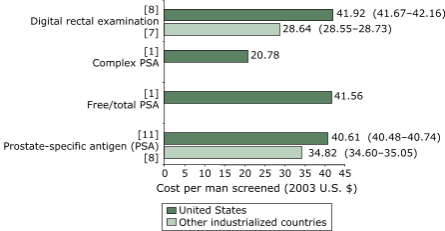
Weighted mean cost (in 2003 U.S. dollars) per man screened for prostate cancer, by type of screening method. Numbers in brackets are the number of studies that reported on each testing method. Numbers in parentheses are 95% confidence intervals. Only U.S. studies reported costs for complex PSA or free/total PSA.

**Figure 3:**
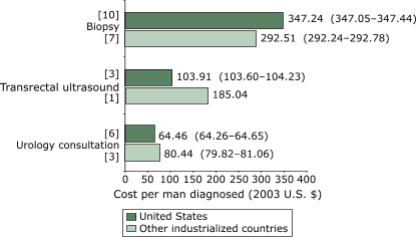
Weighted mean cost (in 2003 U.S. dollars) per man given a diagnostic test for prostate cancer, by diagnostic method. Numbers in brackets are the number of studies that reported on each method. Numbers in parentheses are 95% confidence intervals.

**Figure 4:**
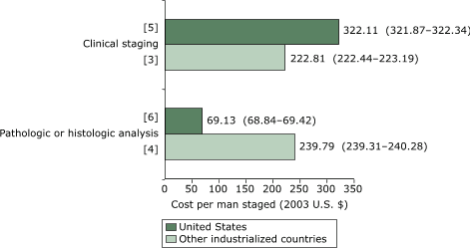
Weighted mean cost (in 2003 U.S. dollars) per man given a clinically staged diagnosis of prostate cancer. Numbers in brackets are the number of studies in each staging method. Numbers in parentheses are 95% confidence intervals.

For studies conducted in the United States, the weighted mean cost per man screened with PSA was $40.61 (95% CI, $40.48–$40.74), compared with $34.82 (95% CI, $34.60–$35.05) for studies conducted in other countries (Figure 2). For diagnostic methods (Figure 3), the mean cost per man was $347.24 (95% CI, $347.05–$347.44) for U.S. studies compared with $292.51 (95% CI, $292.24–$292.78) for non-U.S. studies. For clinical staging methods (Figure 4), the mean cost per man given a clinically staged diagnosis of prostate cancer was $322.11 (95% CI, $321.87–$322.34) for U.S. studies compared with $222.81 (CI, $222.44–$223.19) for non-U.S. studies. The cost per analysis of pathologic or histologic specimens was 3.5 times higher in other countries than in the United States.

**Table:** 

Figure 2 is a bar chart comparing the weighted mean costs per man tested for the United States and for other industrialized countries where data for both were available. The x axis is numbered from $0–$45 and calibrated at intervals of $5. It represents cost per man tested in 2003 U.S. dollars. The y axis consists of four sets of bars labeled as follows:
Prostate-specific antigen (PSA) Free/total PSA Complex PSA Digital rectal examination
The weighted means for prostate specific antigen were as follows:
United States (11 studies): $40.61 ($40.48–$40.74) Other industrialized countries (8 studies): $34.82 ($34.60–$35.05)
The weighted mean for free/total PSA (1 study) was available only for the United States and was $41.56.
The weighted mean for complex PSA (1 study) was available for the United States only and was $20.78.
The weighted means for digital rectal examination were as follows:
United States (8 studies): $41.92 ($41.67–$42.16) Other industrialized countries (7 studies): $28.64 ($28.55–$28.73)

**Table:** 

Figure 3 is a bar chart comparing the weighted mean cost per man, by prostate cancer diagnostic method, for the United States and for other industrialized countries. The x axis is calibrated from $0–$400 in intervals of $50. It represents cost (in 2003 U.S. dollars) per man given a diagnosis of prostate cancer. The y axis consists of three sets of bars labeled as follows:
Urology consultation Transrectal ultrasound Biopsy
The weighted mean cost for urology consultation was:
United States (6 studies): $64.46 ($64.26–$64.65) Other industrialized countries (3 studies): $80.44 ($79.82–$81.06)
The weighted mean cost for transrectal ultrasound was:
United States (10 studies): $347.24 ($347.05–$347.44) Other industrialized countries (7 studies): $292.51 ($292.24–$292.78)

**Table:** 

Figure 4 is a bar chart comparing the weighted mean cost per man for prostate cancer staging for the United States and for other industrialized countries. The x axis is calibrated from $0–$350 in intervals of $50. It represents cost for staging per man in 2003 U.S. dollars. The y axis consists of two sets of bars labeled as follows:
Pathologic or histologic analysis Clinical staging
The weighted mean cost for pathologic or histologic staging was:
United States (6 studies): $69.13 ($68.84–$69.42) Other industrialized countries (4 studies): $239.79 ($239.31–$240.28)
The weighted mean cost for clinical staging was:
United States (5 studies): $322.11 ($321.87–$322.34) Other industrialized countries (3 studies): $222.81 ($222.44–$223.19)

**Figure 5:**
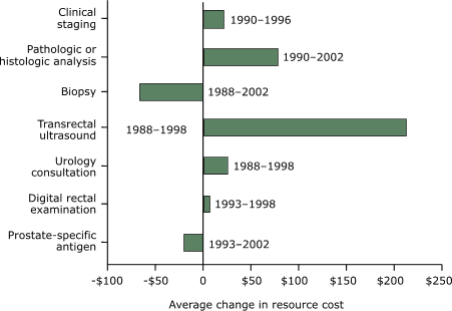
Average changes in resource cost by screening test, diagnostic test, and staging of prostate cancer according to studies conducted in the United States, in 2003 U.S. dollars.

**Figure 6:**
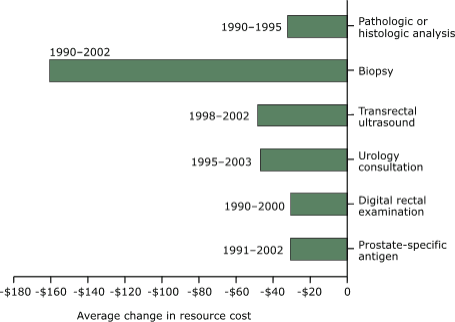
Average changes in resource cost, by screening and diagnostic tests for prostate cancer according to studies conducted in industrialized countries other than the United States, in 2003 U.S. dollars.

In the United States, from 1993 to 2002, the average resource cost of screening with PSA decreased by $20.64 (Figure 5). In addition, from 1988 to 2002 the average resource cost for biopsy decreased by $67.23. However, the average costs for DRE, urology consultation, TRUS, pathologic or histologic analysis of a specimen, and clinical staging increased from the 1990s. In contrast, the average resource costs for all prostate cancer procedures or tests decreased in other industrialized countries (Figure 6). The highest decrease observed was for biopsy ($160.70), and the lowest was for DRE ($30.55).

**Table:** 

Figure 5 is a bar graph showing changes in the cost for various testing, diagnosing, and staging of prostate cancer from 1988 to 2002. The x axis is labeled Average change in resource cost and is calibrated from −$100 to $250. The y axis consists of seven bars labeled with the following data:
Prostate-specific antigen: Cost decreased $20.64 between 1993 and 2002. Digital rectal examination: Cost increased $7.99 between 1993 and 1998 Urology consultation: Cost increased $26.23 between 1988 and 1998. Transrectal ultrasound: cost increased $213.53 between 1988 and 1998. Biopsy: Cost decreased $67.23 between 1988 and 2002. Pathologic or histologic analysis: Cost increased $79.19 between 1990 and 2002. Clinical staging: Cost increased $21.91 between 1990 and 1996.

**Table:** 

Figure 6 is a bar graph showing changes in the cost for various methods of screening for, diagnosing, and staging prostate cancer from 1988 to 2002 in other industrialized countries. The x axis is labeled Average change in resource cost and is calibrated from $0 to −$180. The y axis consists of 6 bars labeled with the following data:
Prostate-specific antigen: Cost decreased $30.97 between 1991 and 2002. Digital rectal examination: Cost decreased $30.55 between 1990 and 2000 Urology consultation: Cost decreased $47.23 between 1995 and 2003. Transrectal ultrasound: cost decreased $48.46 between 1998 and 2002. Biopsy: Cost decreased $160.70 between 1990 and 2002. Pathologic or histologic staging: Cost decreased $32.48 between 1990 and 1995.

### Multivariate sensitivity analyses

The results of the Monte Carlo simulations are in Table 4. The estimated average resource costs of screening for, diagnosing, and clinically staging prostate cancer from studies conducted in the United States and other countries do not differ much from the baseline estimates in Table 3, and the estimated 95% CIs are tight, an indication of the robustness of the baseline results. There is a slight variation in the estimated average resource cost for the histologic analysis of specimens from studies conducted in the other countries compared with the baseline.

For studies conducted in the United States, the estimated median resource costs were $34.54 (interquartile range: $22.91–$49.75) for the PSA test and $234.7 (interquartile range: $152.98–$432.67) for biopsy. These costs vary slightly from the baseline estimates. For studies conducted in other industrialized countries, the median cost of a urology consultation was $92.95 (interquartile range: $74.68–$114.81) and $100.56 (interquartile range: $80.92–$125.25) for TRUS. These costs do not differ substantially from the baseline estimates; however, the median cost for clinical staging in non-U.S. countries differs substantially from baseline with an estimated resource cost of $288.25 (interquartile range: $217.42–$380.62).

## Discussion

Both U.S. and non-U.S. studies estimated the resource costs for screening with PSA and DRE to be greater than $30. We found little variation in the resource costs of screening for prostate cancer between the United States and other industrialized countries; however, the resource costs for diagnostic and staging methods were mixed. Furthermore, we found that the weighted mean cost per man for screening for, diagnosing, and clinically staging prostate cancer varied between the United States and other industrialized countries. Over time, the changes in the average resource costs in U.S. studies were mixed (some increased, and some decreased), but they all decreased in studies conducted in other industrialized countries.

In general, our findings on resource costs of testing methods are consistent with previously published estimates. For example, in 2002 O'Malley and colleagues estimated the resource costs (including cost of the testing method, consumables, and personnel) for screening to be $34 with PSA and $20 with DRE (53). When these costs were adjusted to 2003 U.S. dollars, the estimates were close, with the exception of resource costs for DRE. Compared with the O'Malley study, our estimate of resource costs for DRE is about 31% higher for studies conducted in the United States and 34% higher for studies conducted in other countries. One explanation for this difference could be the differences in the resource measurement used. Because DRE is performed as part of a general physical examination, some studies included the resource cost for a physician visit as part of the cost of DRE (35,36,46). One study estimated that the resource costs attributed to DRE were 13.3% of the total for a physician visit (36), whereas two studies assumed that the resource cost for performing DRE is zero because it is part of the routine annual physical examination (27,34). Furthermore, our estimated resource costs for testing with PSA were within the range of the price ($30–$60) reported in the news media (54).

The resource costs for TRUS, biopsy, and clinical staging were at least 2.3 times higher in the United States than in other countries. This finding is consistent with the general belief that medical technology is more expensive in the United States than in other industrialized nations (55). Although medical technology may cost more in the United States than elsewhere, the resource costs for urology consultation and pathologic or histologic analysis of specimens are at least 1.3 times higher in other countries. For urology consultation, the differences in the resource costs may be explained by differences in the measurement of resource inputs included in the calculation. For example, four of five studies conducted in other countries that met the inclusion criteria for urology consultation included resource costs such as telephone calls, nurse and secretary wages, and hospital visits (42,46,47,50). Other sources of variation could be differences in study settings.

Currently, the evidence is insufficient to determine whether early detection of prostate cancer is cost-effective. The conclusions of the few studies that reported the cost-effectiveness of prostate cancer screening vary widely (12-14). As a result, these economic studies may not inform policy. Several economic studies reported that costs are the major source of variation in differing conclusions for cost-effectiveness of health interventions (56-59). A recent workshop at the Institute of Medicine concluded that poor quality of information on resource costs of screening procedures is a major source of the inconsistency in results among several models of cost-effectiveness of colorectal cancer screening (60). In 1996, the United States Panel on Cost-Effectiveness in Health and Medicine recommended the use of resource-based cost in estimating the cost-effectiveness of health interventions (17). The purpose of this recommendation was to promote transparency and generalizability in the use of economic evaluation results to inform policy. The components of resource costs for early detection of prostate cancer identified in studies included in this paper are patients' travel time, loss of work time, loss of leisure time, transportation cost, physicians' consultation time, other medical staff time, medical supplies, office or room space, equipment, patient recruitment, and other consumables. The range of resource costs included in a particular study depends on the perspective of the study. Because our study took a societal perspective, we chose all identified resource costs. We believe that the pooled resource costs reported in this article may contribute to promoting transparency and generalizability of economic studies on prostate cancer within and among countries.

Screening by PSA remains controversial because of the lack of scientific evidence from clinical trials demonstrating that early detection reduces mortality. In recent years, several variations of the PSA test have been developed that may improve the test's specificity and may reduce the biopsy rate (61-64). These newer testing technologies include free PSA, F/tPSA, cPSA, and PSA density. Among the variations of the PSA test, we were able to find only one U.S. study reporting F/tPSA and cPSA data that met our inclusion criteria (39). The resource costs for these two newer testing methods were $41.56 for F/tPSA and $21.78 for cPSA.

The diagnostic methods considered in our study were TRUS and biopsy. The literature reports that TRUS can be used to screen for prostate cancer, to estimate the size of the prostate, to diagnose cancer, and to guide needle biopsies (65). Although earlier studies evaluated TRUS as a tool for prostate cancer screening (27,31,32,34), it has not been shown to be an effective screening test (66-69). Currently, TRUS is used primarily to image the prostate gland and to guide needle biopsy. For these reasons, we defined TRUS as a diagnostic method. We also assert that this definition is a matter of formality since resource costs were reported separately and not as combined resource costs of diagnosing prostate cancer.

Biopsy is currently the gold standard method for diagnosing prostate cancer (66). Several biopsy procedures can confirm the diagnosis of prostate cancer (7,49,64). Therefore, we combined the resource costs of these biopsy procedures because many of the studies reviewed reported aggregated resource cost for each procedure.

The final method in the evaluation and management of prostate cancer, clinical staging, is important because it is the first step in determining prognosis and because it guides treatment decisions for men with an established diagnosis of cancer. In most of the studies reviewed, the researchers used different types of clinical staging procedures. For example, Gottlieb et al used CT, MRI, and radionuclide bone scan (36). As with biopsy, some researchers reported combined resource costs for clinical staging. Therefore, the resource cost we report is a combination of resource costs for various procedures.

Our study has limitations. Most of these are primarily tied to the limitations in the studies we reviewed. First, the reported resource costs were pooled from studies conducted with different populations and in different settings and should be interpreted with caution. However, we conducted appropriate sensitivity analyses using Monte Carlo simulation to assess the robustness of the baseline results. Second, PSA is used as a screening test, a diagnostic tool, and a biological marker to follow the progress of disease in men with prostate cancer, but we did not distinguish reported resource costs for PSA among its uses. We are not sure whether this lack of distinction may have underestimated or overestimated the reported resource costs for PSA. Third, for some studies it was difficult to separate the resource cost of performing DRE from that of the physician visit. Because of the strong interaction, we may have overestimated resource costs for DRE. Fourth, because reported resource costs for performing biopsy and clinical staging represented combined resource costs from several procedures, they should be interpreted with caution. Furthermore, reported resource costs for performing TRUS or biopsy did not include the cost of complications resulting from these procedures. It has been reported that complication costs are directly correlated to the biopsy rate (70). Gustafsson et al suggest that the resource costs associated with complications arising from TRUS or biopsy should be reported separately from those for diagnostic procedures (46) because the cost of complications depends on the number of infections, which ranges from 5% to 6%, and their severity (71,72). Fifth, for studies conducted in other countries, we are not sure if the resource costs reported by some authors were costs or charges. If some resources are charges rather than costs, then the pooled resource costs presented here for studies conducted in other countries may be lower than we report.

Finally, identifying and measuring all the resources used in screening for, diagnosing, and clinically staging prostate cancer is a time-consuming and expensive process. In many situations, such detailed evaluations may not be practical. Therefore, the resource cost estimates reported will invariably diverge from the microcosting approach recommended by the U.S. Panel on Cost-Effectiveness in Health and Medicine (17).

To examine the policy implications of resource cost estimates reported here for decision making, we assumed the worst-case scenario in which 50 million men aged 40 to 74 years in the United States (73) receive a PSA test annually. Using the baseline societal resource cost estimate of $37.23 per test, this would translate approximately into an undiscounted $1.86 billion per year. Of course, this estimate may not be realistic given that not all eligible men in the population would be tested annually. Similarly, it may be difficult to justify screening men aged 40 to 49 years in the entire population unless there is a family history of prostate cancer or the man is of African American descent. This example illustrates how the results presented in this paper may be used by policy makers in making decisions regarding resource allocation for prostate cancer. Similarly, researchers may use the resource cost estimates presented in this paper as one of the input variables in estimating the cost-effectiveness of screening for prostate cancer and detecting it early.

The effectiveness of early detection in reducing the mortality associated with prostate cancer is still a matter of debate. With limited health resources, investing in early-detection services for prostate cancer will require an understanding of resource costs used in estimating the economic cost of this disease. Therefore, realistic resource cost estimates are necessary to calculate meaningful cost-effectiveness estimates for prostate cancer screening, diagnosing, and staging. Our analysis may benefit health policy makers charged with allocating resources to prostate cancer.

## Figures and Tables

**Table 1:** Characteristics of Prostate Cancer Studies Reviewed, United States and Other Industrialized Countries, 1980–2003

Study	Base Year^a^	No. Men Tested	No. Biopsies Performed	No. Cancers Detected	Age of Men Tested, y	Study Setting

Studies conducted in the United States						
Torp-Pedersen et al (26)	1988	784	93	30	Not reported	Multi-institutional study in the United States
Optenberg et al (14)	1988	17,496,288	NR	24,229	50-70	NR
Babaian et al (27)	1992	1,860	436	170	63	M.D. Anderson Cancer Center
Dorr et al (28)	1992	19,139,490	3,423,842	1,047,695	50-75	NR
Kramer et al (29)	1990	18,856,430	NR	654,305	50-74	NR
Littrup et al (30)	1992	2,425	271	129	55-70	ACS-NPCDP study
Abramson et al (31)	1992	564	119	18	40-75	Baptist Medical Center, Jacksonville, Florida
Benoit et al (32)^b^	1992	5,340	825	177	50-69	Multicenter study
		8,529	639	209	50-70	Washington University study
Krahn et al (33)	1992	NR	NR	NR	50-70	NR
Kantrowitz et al (34)	1995	1,219	23	12	50-65	Polaroid Corp, work site
OTA (35)	1992	18,300	NR	626	65-75	NR
Gottlieb et al (36)	1995	NR	NR	NR	50-70	NR
Snyder et al (37)	1995	788	52	1	40-75	Zeneca Pharmaceutical Corp, work site
Weinrich et al (38)	2001	892	23	10	40-70	Work sites and churches in 11 South Carolina counties
Ellison et al (39)	2001	2,138	NR	620	40-75	Multiple sites (e.g., UroCor Laboratories and Bayer Diagnostics)

Studies conducted in other industrialized countries						

Carlsson et al (44)	1987	1,163	44	13	50-69	Municipal residence in Norrkoping, Sweden
Pedersen et al (45)	1989	1,163	45	13	50-69	Norrkoping, Sweden
Chadwich et al (48)	1990	472	29	7	55-70	Large city general practice, United Kingdom
Green et al (40)	1992	33,627	NR	NR	50-70	Setting not reported, Canada
Labrie et al (41)	1993	7,350	761	252	45-80	Laval Univ Prostate Cancer Detection Program, Canada
Gustafsson et al (46)	1990	1,782	413	65	55-70	Soder Hospital, Stockholm, Sweden
Chamberlain et al (49)	1995	NR	NR	NR	NR	Setting not reported, United Kingdom
Holmberg et al (47)	1996	1,492	NR	34	50-69	Norrkoping, Sweden
Perkins et al (51)	1995	474	NR	NR	40-79	New South Wales, Australia
Krahn et al (42)	1995	533,402	45,873	9,074	50-74	National survey of Canadian men, population-based
Candas et al (43)	1998	9,296	913	282	45-80	Quebec City, Canada
Kosuda et al (52)	2001	NR	NR	1294	42-93	Multicenter study of five working group hospitals, Japan
Donovan et al (50)	2001	7,383	592	165	50-69	Setting not reported, United Kingdom

NR indicates not reported; ACS–NPCDP American Cancer Society–National Prostate Cancer Detection Project; OTA, Office of Technology Assessment.

a The year in which the data used in the study were collected.

b This study was conducted in more than one setting. We report the figures for each setting separately. The multicenter study was done at the University of California and in Canada.

**Table 2:** Standardized Resource Costs for Prostate Cancer Screening, Diagnosing, and Staging, in U.S. Dollars, United States and Other Industrialized Countries, 1980–2003

Study	Testing Methods^a^				Diagnostic Methods			Staging Methods	

	PSA	F/t PSA	cPSA	DRE	Urology Consult^b^,^c^	TRUS	Biopsy^d^	Pathologic or Histologic^c^	Clinical^e^

Studies conducted in the United States^f^									
Torp-Pedersen et al (26)^g^	NR	NR	NR	NR	44.48	211.51	300.46197.67	NR	NR
Optenberg et al (14)	NR	NR	NR	NR	NR	NR	245.57	66.27	676.94
Babaian et al (27)	45.38	NR	NR	0.00	NR	147.06	105.04	84.04	NR
Dorr et al (28)	57.68	NR	NR	NR	NR	280.55	413.65	NR	197.86
Kramer et al (29)	23.30	NR	NR	41.94	NR	NR	139.81	NR	507.06
Littrup et al (30)	39.33	NR	NR	39.33	NR	196.65	655.50	NR	910.27
Abramson et al (31)	32.78	NR	NR	32.78	98.33	131.10	150.77	131.10	NR
Benoit et al (32)	24.67	NR	NR	4.20	58.51	71.90	105.46	45.77	NR
Krahn et al (33)	13.11	NR	NR	0.00	—	159.94	308.09	81.28	1,067.15
Kantrowitz et al (34)	35.50	NR	NR	12.58	156.04	488.84	364.50	NR	NR
Office of Tech-nology Assess-ment (35)	24.84	NR	NR	26.05	39.60	71.38	158.96	105.04	1,097.53
Gottlieb et al (36)	34.94	NR	NR	NR	NR	NR	489.20	NR	698.85
Snyder et al (37)	54.54	NR	NR	35.78	76.25	445.74	1,923.72	NR	NR
Weinrich et al (38)	77.18	NR	NR	61.48	65.18	404.35	549.19	NR	NR
Ellison et al (39)	20.78	41.56	20.78	NR	NR	NR	181.83	145.46	NR

Studies conducted in other industrialized countries^h^									

Carlsson et al (44)	NR	NR	NR	66.66	NR	NR	240.00	128.22	NR
Pedersen et al (45)	NR	NR	NR	40.70	NR	NR	194.60	NR	NR
Chadwich et al (48)	46.53	NR	NR	NR	NR	NR	31.64	59.83	NR
Green et al (40)	25.76	NR	NR	NR	NR	NR	NR	NR	NR
Labrie et al (41)	24.67	NR	NR	24.67	NR	NR	197.40	NR	NR
Gustafsson et al (46)	69.00	NR	NR	53.41	147.84	NR	226.55	95.74	NR
Chamberlain et al (49)	36.54	NR	NR	21.94	NR	NR	NR	NR	NR
Holmberg et al (47)	14.67	NR	NR	16.13	101.56	NR	123.67	NR	146.74
Perkins et al (51)	17.61	NR	NR	21.68	55.61	87.37	NR	NR	NR
Krahn et al (42)	35.69	NR	NR	NR	79.57	NR	298.51	241.13	168.79
Candas et al (43)	23.13	NR	NR	23.13	NR	185.04	115.65	NR	NR
Kosuda et al (52)	15.56	NR	NR	NR	NR	38.91	56.60	NR	603.67
Donovan et al (50)	NR	NR	NR	NR	100.61	NR	NR	NR	NR

NR indicates not reported; TRUS, transrectal ultrasound

a PSA indicates prostate-specific antigen; F/t PSA, free/total PSA; cPSA, complex PSA; DRE, digital rectal examination.

b Any consultation or referral to a urologist, clinical oncologist, or any other specialist.

c *Urology consultation* and *pathologic or histologic analysis of specimen* are process measures, not methods.

d Resource costs of biopsy include costs for core-needle, TRUS-guided fine-needle aspiration, needle, and transrectal needle.

e Resource cost of clinical staging procedures includes computed tomography (CT), magnetic resonance imaging (MRI), radionuclide bone scan, pelvic lymph node excision and analysis, and pelvic echography.

f All resource costs were standardized to 2003 U.S. dollars using the Consumer Price Index (CPI) for the year that cost data were collected. In addition, all costs that were originally presented as charges (prices), which may not reflect the true resource cost of providing prostate cancer-related services, were converted to cost by using a cost-to-charge ratio.

g The $300.46 figure is the real cost of performing biopsies at institutions in six states: New York, California, Alabama, Massachusetts, Arizona, and Texas. The researchers found that the the charges at another institution in southeastern Michigan were reported as $197.67. The two figures cannot be combined since charges are not the same as costs.

h Canada, Australia, Japan, Sweden, and the United Kingdom. All resource costs were standardized to 2003 U.S. dollars using the country-specific Consumer Price Index for the year that cost data were collected and the Purchasing Power Parity method.

**Table 3:** Pooled Resource Costs for Prostate Cancer Screening, Diagnosing, and Staging, in U.S. Dollars, United States and Other Industrialized Countries, 1980–2003

Method [No. Studies Using Method]	Pooled Resource Costs,2003 U.S. $			Probability Distribution^a^

	Baseline	Min	Max	

Studies conducted in the United States				
**Screening**				
Prostate-specific antigen (PSA) [13]	37.23	13.11	77.18	Triangular
Free/total PSA [1]	41.56	20.78	62.34	Triangular
Complex PSA [1]	20.78	10.39	41.56	Triangular
Digital rectal examination [8]	31.77	4.20	61.48	Normal
**Diagnostic **				
Urology consult b,c [7]	76.91	39.60	156.04	Normal
Transrectal ultrasound [11]	237.18	71.38	488.84	Normal
Biopsy [15] d	393.09	105.04	1,923.72	Invgauss
**Staging**				
Pathologic or histologic [7]^c^	94.14	45.77	145.46	Log normal
Clinical staging [7]^e^	736.52	197.86	1,097.53	Normal

Studies conducted in other industrialized countries^f^				
**Screening**				
Prostate-specific antigen (PSA) [10]	30.92	15.56	69.00	Normal
Free/total PSA [0]	NR	NR	NR	NR
Complex PSA [0]	NR	NR	NR	NR
Digital rectal examination [8]	33.54	16.13	66.66	Normal
**Diagnostic**				
Urology consultation [5] b,c	97.04	55.61	147.84	Log normal
Transrectal ultrasound [3]	103.77	38.91	185.04	Triangular
Biopsy [9] d	164.96	31.64	298.51	Normal
**Staging**				
Pathologic or histologic [4]^c^	131.23	59.83	241.13	Uniform
Clinical staging [3] e	306.40	146.74	603.67	Triangular

NR indicates not reported; TRUS, transrectal ultrasound

a Probability distributions used in conducting multivariate sensitivity analysis.

b Urology consult was defined as any consultation or referral to a urologist, clinical oncologist, or any other specialist.

c *Urology consultation* and *pathologic or histologic analysis of specimen* are process measures, not methods.

d Resource costs of biopsy include costs for core needle, TRUS-guided, fine-needle aspiration, needle, and transrectal needle.

e Resource costs of clinical staging procedures include computed tomography, magnetic resonance imaging, radionuclide bone scan, pelvic lymph node excision and analysis, and echography.

f Canada, Australia, Japan, Sweden, and the United Kingdom.

**Table 4:** Multivariate Sensitivity Analyses from Monte Carlo Simulations, Estimated Average Resource Costs for Methods of Prostate Cancer Screening, Diagnosing, and Staging, United States and Other Industrialized Countries, 1980–2003

Method	Studies in the United States			Studies in Other Industrialized Countries^a^		

	Mean (95% CI)	Median	InterquartileRange	Mean (95% CI)	Median	InterquartileRange

	2003 $					
**Screening**						
Prostate-specific antigen (PSA)	37.55 (36.48-38.62)	34.54	(22.91-49.75)	30.92 (29.87-31.97)	30.91	(19.56-42.24)
Free/total PSA	41.56 (41.03-42.09)	41.56	(35.47-47.63)	NR	NR	NR
Complex PSA	24.24 (23.64-24.64)	23.56	(19.39-28.83)	NR	NR	NR
Digital rectal examination	31.77 (30.67-32.88)	31.73	(19.73-43.76)	33.56 (32.43-34.69)	33.51	(21.26-45.79)
**Diagnostic**						
Urology consultation^b^,^c^	76.91 (74.43-79.39)	76.84	(49.78-103.89)	97.06 (95.12-99.00)	92.95	(74.68-114.81)
Transrectal ultrasound	237.15 (227.97-246.33)	236.92	(137.12-336.85)	103.77 (101.89-105.66)	100.56	(80.92-125.25)
Biopsy^d^	392.83 (364.61-421.04)	234.70	(152.98-432.67)	164.92 (159.42-170.42)	164.76	(105.11-224.58)
**Staging**						
Pathologic or histologic^c^	94.14 (92.07-96.21)	90.74	(70.33-114.14)	139.04 (137.24-140.84)	138.97	(113.85-164.13)
Clinical^e^	736.56 (716.66-756.45)	736.24	(519.11-952.25)	306.4 (299.88-312.93)	288.25	(217.42-380.62)

NR indicates not reported; TRUS, transrectal ultrasound

a Canada, Australia, Japan, Sweden, and the United Kingdom.

b *Urology consultation *was defined as any consultation or referral to a urologist, clinical oncologist, or any other specialist.

c Urology consult and pathologic or histologic analysis of specimen are process measures, not methods.

d Resource costs of biopsy include costs for core needle, TRUS-guided, fine-needle aspiration, needle, and transrectal needle.

e Resource cost of clinical staging procedures included computed tomography, magnetic resonance imaging, radionuclide bone scan, pelvic lymph node excision and analysis, and pelvic echography.
